# Perceptions and practices regarding the process of obtaining informed consent from surgical patients at a tertiary care hospital

**DOI:** 10.1016/j.amsu.2021.103195

**Published:** 2021-12-22

**Authors:** Muhammad Asharib Arshad, Naureen Omar, Zaid Amjad, Khalid Bashir, Muhammad Irfan, Irfan Ullah

**Affiliations:** a FMH College of Medicine and Dentistry, Lahore, Pakistan; b Department of Emergency Medicine, Hamad Medical Corporation, Qatar; c Internal Medicine, Hayatabad Medical Complex, Peshawar, Pakistan; d Kabir Medical College, Gandhara University, Peshawar, Pakistan

**Keywords:** Informed consent, Bioethics, Professionalism

## Abstract

**Background:**

Proper informed consent is essential for patients to have sound knowledge about the indication, risks, and benefits of a proposed surgical procedure. The study aim was to assess the perceptions of postoperative patients about the informed consent process and identify various influential factors in a tertiary care hospital.

**Methods:**

A cross-sectional study was conducted from February to August 2018 at a tertiary care hospital in Lahore, Pakistan. A validated questionnaire was used to conduct interviews of 101 patients planning to undergo elective surgery after fulfilling all ethical considerations. A purposive sampling technique was employed to enroll and the data analysis was performed by using SPSS version 23.

**Results:**

Out of total 101 patients, 50 (49.5%) of them were males and the mean age of total sample was 36.98 ± 14.23 years. The majority 92 (91.1%) considered informed consent to be important and that it did not influence their surgical decision 85 (84.2%). Consent was obtained by the consulting surgeon from 41 (40.6%) patients and by the residents/house officer from 60 (59.4%) patients. Fifteen (14.8%) patients signed the consent form themselves, and 86 (85.1%) relatives of patients signed. Ninety-eight (97.0) patients were told about indications of the surgery, and 54 (53.5%) were told about possible complications. Seventy-five (74.3%) patients were informed about alternatives to surgery. Significant reasons for not signing were language (p = 0.03), educational status (p = 0.002), and not being informed by relatives before signing (p = 0.02).

**Conclusion:**

The patients had adequate knowledge about the process of informed consent and considered it important. Factors identified as barriers to signing the consent form by the patients themselves included language, better educational status, and not being asked by relatives. It is imperative to involve the patients in the process of consent, especially in signing by them or in their presence by their surrogate.

## Highlights

• Informed consent is the basic right of the patient to express acceptance or rejection based on the information provided by the physician about the proposed medical treatment.
• Patients had adequate knowledge about the process of informed consent and considered it important in developing countries.
• Factors identified as barriers in signing of consent form by patients themselves included language, better educational status and not being asked by relatives.
• A higher proportion of patients are satisfied with the process of informed consent.

## 1 Introduction

Biomedical ethics is an aspect of medicine that concerns morality, which is important because it builds trust between the doctor and patient. Doctors have a moral obligation to ensure that all information about the patient's conditions and any proposed procedures are communicated and that the patient is not harmed [1,2]. However, biomedical ethics is a constantly evolving field, and doctors must continually keep up with new developments related to their branch of medicine [3]. The foundation of medical ethics is based on the following key principles: informed consent, privacy, and confidentiality [2].

Informed consent is the process whereby patients express their acceptance or refusal to undergo the proposed medical intervention on the basis of information provided by a health care professional regarding the nature and potential consequences of a proposed treatment [4]. The aim of informed consent is to ensure that patients can autonomously be involved in decisions related to their health care [1]. This process involves not only the patient's autonomy, but also disclosure of complete and correct information. Doctors are to elaborate in detail about the diagnostic, therapeutic, and prognostic factors of the optimal treatment and how it will specifically benefit the patient [5]. A study conducted in Karachi, Pakistan determined that informed consent was obtained from 66.4% of the patients [6].

There has been some recent progress in Pakistan toward creation of guidelines for health care providers conducting medical practice and research. The Pakistan Medical and Dental Council, which is the statutory regulatory authority of Pakistan, has developed a code of ethics for medical practitioners, although it has not been consistently implemented partly because cultural values are an obstacle in practicing medical ethics in Pakistan [7]. Earlier qualitative studies have shown that most health professionals think that after giving detailed information to the patient, they do not consider obtaining formal consent to be essential [8,9].

Social media has a great impact on our daily lives and has been one source of increased awareness of patients regarding the importance of informed consent and autonomy of patients, thus making them more inquisitive about their treatment, potential complications, and expected benefits of the surgery [3].

Therefore, there is an urgent need to understand the implications of informed consent from a patient's perspective [10]. The study aim was to assess the perceptions of postoperative patients about the process of informed consent and to identify various factors that influence this process at a private tertiary care hospital in Lahore, Pakistan.

## 2 Methods

### 2.1 Participants and study design

A hospital-based cross-sectional study was conducted between February 2018 and August 2018. The study was conducted at a 500 beds private multi-specialty hospital based in Lahore, Pakistan. Approximately 8400 patients undergo operations every year. There are six trainees working in the surgical department. Patients undergoing elective surgery who were adults [>18 years of age] were enrolled, and we selected an equal number of male and female participants to avoid sex bias. Patients with a hearing impairment, any psychiatric disorder, or who were experiencing pain and/or admitted under emergent conditions were not included.

### 2.2 Simple size

A purposive non-probability, convenience sampling technique was employed to enroll a total of 101 patients on the basis of the monthly patient input of the operation theater (700). The confidence interval was 95%, the study power was 80%, and the alpha value was 5% per the following formula:

n=z1−α22P(1−p)d2

### 2.3 Study procedure

The study was approved by the Institutional Review Board of our private tertiary care hospital (07-2019-IRB-641-M). The data was collected on a slightly modified and pre-tested validated Performa (used with permission) from the patients who fulfilled the inclusion criteria after providing consent [10]. The form comprised questions about the demographic status, perception, and factors influencing the process of informed consent of the patients. Principal investigators collected the data from the patients planning to undergo surgery and respected the anonymity of the patients and institution. Our study is fully compliant with the STROCSS criteria [11]. This study was registered with research registry with the registration number (researchregistry7112).

### 2.4 Statistical analysis

Mean and standard deviation were calculated for quantitative variables while frequency and percentages were calculated for qualitative variables. Chi-Square test was applied for categorical variables. Data were analyzed by using the statistical package for social science (SPSS) version 22.

## 3 Results

The mean age of the patients (n = 101) was 36.98 ± 14.23 years (range: 18–82 years). There were 50 (49.5%) males and 51 (50.5%) females; 79 (78.2%) were married and 22 (21.8%) were single. Only 15 (14.9%) patients could not read and write, whereas 12 (11.9%) had been educated at the primary level, 23 (22.8%) at the secondary level, and 51 (50.5%) at or above the intermediate level. Fifteen (14.9%) patients signed their consent form themselves, whereas nine (8.9%) were signed by parents, 38 (37.6%) by spouses, 16 (15.8%) by their children, and 23 (22.8%) by their siblings. Of the 15 (14.8%) patients who signed themselves, 12 (80%) were males and three (20%) were females, but 86 (85.1%) of the consent forms were signed by the relatives of the patients, of which 38 (44.18%) were males and 48 (55.81%) were females.

Table 1 shows the perceptions of patients regarding the process of informed consent. Sixty (59.4%) patients were given information about the surgery by the residents, 57 (56.4%) patients provided consent in the ward, and 88 (87.1%) patients did not think that they had been influenced by anyone to proceed with surgery. Ninety-two (91.1%) of the patients realized it was important to provide informed consent before any surgery. Fifty-five (54.5%) of the patients were aware of the medico-legal significance of informed consent. Seventy-three (72.3%) of the patients preferred detailed information before proceeding with any surgery. Ninety-one (90.1%) patients were satisfied with the information provided, 93 (92.1%) patients thought their confidentiality was maintained, and 98 (97%) patients considered that their enquiries were appropriately responded to by their doctor.

**Table 1:** Perceptions of patients and relatives who signed the consent form for surgery about the process of informed consent.

Characteristics of consent	Total (n = 101)	Males (n = 50, 49.5%)	Female (n = 51, 51.5%)	p value
**Informed Consent: Who explained the information**				0.233
Consultant Surgeon	41 (40.6)	18 (17.8)	23 (22.8)	
Resident/House officer	60 (59.4)	32 (31.7)	28 (27.7)	
**Informed Consent: Where was the information provided**				0.003
Operating theatre	27 (26.7)	6 (5.9)	21 (20.8)	
Ward	50 (49.5)	31 (30.7)	19 (18.8)	
Clinic	24 (23.8)	13 (12.9)	11 (10.9)	
**Informed Consent: Where was the consent taken**				0.006
Operating theatre	36 (35.6)	11 (10.9)	25 (24.8)	
Ward	57 (56.4)	36 (35.6)	21 (20.8)	
Clinic	8 (7.9)	3 (3.0)	5 (5.0)	
**Informed consent: influenced your decision to proceed with surgery**				0.220
Yes	16 (15.8)	6 (5.9)	10 (9.9)	
No	85 (84.2)	44 (43.6)	41 (40.6)	
**Informed consent: Decision to proceed with surgery was influenced by others**				0.235
No	88 (87.1)	45 (44.6)	43 (42.6)	
Yes, family/friends	2 (2.0)	0 (0.0)	2 (2.0)	
Yes, doctor	11 (10.9)	5 (5.0)	6 (5.9)	
**Informed consent: Important before any surgery**				0.234
Yes	92 (91.1)	44 (43.6)	48 (47.5)	
No	9 (8.9)	6 (5.9)	3 (3.0)	
**Informed Consent: Knowledge of medico-legal significance of informed consent**				0.457
Yes	46 (45.5)	22 (21.8)	24 (23.8)	
No	55 (54.5)	28 (27.7)	27 (26.7)	
**Informed consent: Amount of information preferred**				0.052
Limited	28 (27.7)	18 (17.8)	10 (9.9)	
Detailed	73 (72.3)	32 (31.7)	41 (40.6)	
**Amount of information preferred if going for same surgery**				0.005
Limited	37 (36.6)	25 (24.8)	12 (11.9)	
Detailed	64 (63.4)	25 (24.8)	39 (38.6)	
**Informed Consent: Satisfied with information provided**				0.383
Yes	91 (90.1)	46 (45.5)	45 (44.6)	
No	10 (9.9)	4 (4.0)	6 (5.9)	
**Informed Consent: Are you in the favor of the process**				0.252
Yes	99 (98.0)	50 (49.5)	49 (48.5)	
No	2 (2.0)	0 (0.0)	2 (2.0)	
**Informed Consent: Was your confidentiality assured**				0.141
Yes	93 (92.1)	48 (47.5)	45 (44.6)	
No	8 (7.9)	2 (2.0)	6 (5.9)	
**Informed Consent: Was your privacy maintained**				0.007
Yes	80 (79.2)	45 (44.6	35 (34.7)	
No	21 (20.8)	5 (5.0)	16 (15.8)	
**Informed Consent: Were your enquires replied by the doctor**				0.492
Yes	98 (97.0)	48 (47.5)	50 (49.5)	
No	3 (3.0)	2 (2.0)	1 (1.0)	

Table 2 presents information about the attitudes of the patients and their relatives who signed the consent form toward the important clauses of the consent form. Eighty-one (80.2%) and 98 (97%) patients were informed about the nature and indications of surgery, respectively. Fifty-four (53.5%) and 53 (52.5%) patients were informed about the possible complications of surgery and length of hospital stay after surgery, respectively. Seventy-five (74.3%) patients were informed about alternatives to surgery. Seventy-eight (77.2%) and 85 (84.2%) patients were told about the possible complications of the disease if the surgery was not performed and the benefits of the surgery, respectively. Eighty-three (82.2%) and 92 (91.1%) patients were informed about the type of anesthesia or given an opportunity to ask questions, respectively.

**Table 02:** Attitudes of patients and relatives who signed the surgery consent form towards the important clauses of the consent form.

Characteristics of consent	Total (n = 101)	Self-signed (n = 15, 14.85%)	Relative signed^a^ (n = 86, 85.14%)	p value
**Informed about nature of surgery**				0.142
Yes	81 (80.2)	10 (66.7)	71 (82.6)	
No	20 (19.8)	5 (33.3)	15 (17.4)	
Don't know	0 (0.0)	0 (0.0)	0 (0.0)	
**Informed about indications for surgery**				0.386
Yes	98 (97.0)	14 (93.3)	84 (97.7)	
No	3 (3.0)	1 (6.7)	2 (2.3)	
Don't know	0 (0.0)	0 (0.0)	0 (0.0)	
**Informed about possible complications of surgery**				0.005
Yes	54 (53.5)	3 (20.0)	51 (59.3)	
No	47 (46.5)	12 (80.0)	35 (40.7)	
Don't know	0 (0.0)	0 (0.0)	0 (0.0)	
**Informed about length of hospital stay after surgery**				0.213
Yes	53 (52.5)	5 (33.3)	48 (55.8)	
No	47 (46.5)	10 (66.7)	37 (43.0)	
Don't know	1 (1.0)	0 (0.0)	1 (1.2)	
**Informed About Alternatives to surgery**				0.577
Yes	75 (74.3)	11 (73.3)	64 (74.4)	
No	26 (25.7)	4 (26.7)	22 (25.6)	
Don't know	0 (0.0)	0 (0.0)	0 (0.0)	
**Informed about possible complications if surgery was not done**				0.007
Yes	78 (77.2)	7 (46.7)	71 (82.6)	
No	21 (20.8)	8 (53.3)	13 (15.1)	
Don't know	2 (2.0)	0 (0.0)	2 (2.3)	
**Informed about expected benefits of surgery**				0.013
Yes	85 (84.2)	9 (60.0)	76 (88.4)	
No	16 (15.8)	6 (40.0)	10 (11.6)	
Don't know	0 (0.0)	0 (0.0)	0 (0.0)	
**Informed about type of anesthesia**				0.779
Yes	83 (82.2)	13 (86.7)	70 (81.4)	
No	17 (16.8)	2 (13.3)	15 (17.4)	
Don't know	1 (1.0)	0 (0.0)	1 (1.2)	
**Given opportunity to ask questions**				0.399
Yes	92 (91.1)	13 (86.7)	79 (91.9)	
No	9 (8.9)	2 (13.3)	7 (8.1)	
Don't know	0 (0.0)	0 (0.0)	0 (0.0)	

a Relatives: parent, spouse, sibling, other relative.

Table 3 presents the factors associated with patients who did not provide informed consent before surgery themselves. Only eighteen (17.8%) patients considered language to be a barrier in signing the consent form themselves. Only 36 (35.6%) patients thought that their relative who signed the informed consent had better educational status. Only 28 (27.7%) patients considered the use of medical terminology to be a factor associated with not signing the consent form themselves. Sixteen (15.8%) patients thought that the time allotted was insufficient to sign the consent form, and the same number of people thought that consent was obtained at an inappropriate time. Twenty-three (22.8%) patients responded that they were not asked by relatives to sign the consent form before the relatives signed to consent. Statistical analysis of educational status revealed that language (p = 0.03), better educational status (p = 0.002), and not being asked by relatives before signing their consent form (p = 0.02) were significant factors associated with the patients not signing their consent form themselves.

**Table 3:** Factors associated with patients not signing the surgery informed consent form for themselves.

Variables	Total (n = 101)	Illiterate (n = 15, 14.85%)	Literate^a^ (n = 86, 85.14%)	p value
**Language Factor**				0.034
Yes	18 (17.8)	6 (5.9)	12 (11.9)	
No	83 (82.2)	9 (8.9)	74 (73.3)	
**Better Educational Status**				0.002
Yes	36 (35.6)	8 (7.9)	28 (27.7)	
No	65 (64.4)	7 (6.9)	58 (57.4)	
**Insufficient Time Allowed**				0.721
Yes	16 (15.8)	3 (3.0)	13 (12.9)	
No	85 (84.2)	12 (11.9)	73 (72.3)	
**Medical Terminology Used**				0.643
Yes	28 (27.7)	4 (4.0)	24 (23.8)	
No	73 (72.3)	11 (11.9)	62 (61.4)	
**Inappropriate Timing**				0.416
Yes	16 (15.8)	2 (2.0)	14 (13.9)	
No	85 (84.2)	13 (12.9)	72 (71.3)	
**Not Informed**				0.022
Yes	23 (22.8)	2 (2.0)	21 (20.8)	
No	78 (77.2)	13 (12.9)	65 (64.4)	

a Literate: primary, secondary, intermediate and above.

## 4 Discussion

Informed consent, a core essential of medical ethics and professionalism, is a process in which a patient makes an autonomous decision to choose or reject any medical intervention after considering complete information about the nature of intervention, its indications, other treatment options available, its benefits and complications, and consequences if the intervention is not conducted [1,3]. The latest trends and usage of internet and electronic media have increased the demands of patients for complete information prior to intervention [6].

In our study, only a few participants signed the consent form themselves, which is in contrast to another study conducted in Karachi in which more than half of the participants signed the consent form themselves [12]. Studies have reported that relatives play a significant role in the signing of the informed consent on behalf of the patients in other countries as well [12,13].In our study, information about the surgery was received by 24 (23.8%) patients in the clinic followed by 50 (49.5%) in the ward. Fifty-seven (56.4%) of the consent forms were signed in the ward. According to the guidelines of the General Medical Council of the United Kingdom, this location allows more time for the patient to contemplate of the information provided before deciding [14]. According to the guidelines of the American College of Surgeons it is not allowed to make promises or give any sort of guarantee about the outcomes of the procedure. It is also prohibited to exaggerate the potential benefits of the surgical procedure [15].

In our study, 41 (40.6%) patients were informed about the surgery by the operating consultants. In a study conducted by F Wood et al., in 2016, they reported the perspectives of the doctors about the process of informed consent. In that study most of the doctors realized that it is the responsibility of the operating surgeon to obtain informed consent [16]. That being said, most of the senior doctors agreed that this responsibility was passed on to the junior doctors [16].

The majority of the patients thought that they were not influenced by anyone to proceed with surgery. Almost half of the patients were aware of the medico-legal significance of the process of informed consent. According to a fact sheet of the Canadian Medical Protective Association for a 5-year period, 65% of medico-legal actions related to informed consent were taken by surgical patients, and only 21% of these legal cases were decided in favor of the surgeon [17]. The majority of the patients in our study approved of the process of informed consent, with more than half of the patients having a preference for detailed information before undergoing surgery. Almost all patients thought that their confidentiality was assured and privacy maintained. Our results were better than those of a study conducted in Karachi that showed a smaller percentage of patients whose confidentiality was assured, which may be attributed to the fact that we conducted the study in a private tertiary care setup [12].

Regarding the attitudes of the patients and relatives who signed the consent form, there were significant differences only in a few of the important clauses in the informed consent for surgery, such as being informed about the possible complications of surgery (p = 0.005), the possible complications if the surgery was not performed (p = 0.007), and about the expected benefits of the surgery (p = 0.013). In our study, 86.7% of the patients who signed their consent form themselves were informed about the type of anesthesia, compared with 81.4% of the relatives who signed the consent form. Previous research studies have shown that knowledge of the important aspects of the process of informed consent was not only lacking in patients but in health professionals as well [18]. Ninety-two (91.1%) patients were given the opportunity to ask questions. Approximately half of the patients knew about the possible complications of surgery and the length of hospital stay after surgery. Our findings are supported by those of previous studies, such as the finding that the risk of an intervention or treatment is usually not told to patients in our society. Doctors prefer to give information about the benefits of surgery and do not tell about possible complications of the procedure, which goes against bioethics because it is necessary to be informed about the common risks of the procedure or treatment [8,19]. More than 70% of our patients and relatives were informed about the nature of the surgery, its indications, alternatives, complications if not performed, benefits, and type of anesthesia administered and were given the opportunity to ask questions. Compared with the findings of another study in 2014 by Karachi, our results regarding the attitudes of patients and relatives toward the important clauses of the consent form were better because a higher percentage of patients and relatives were informed [12].

The barriers associated with patients not signing consent form themselves identified in our study were language, better educational status, and not being informed by relatives before signing the consent form. A previous study conducted in Iran in 2017 found that medical terminology and educational status were barriers to signing the consent form themselves [20]. It has also been reported that literacy affects a patient's understanding of the procedure [21]. A surrogate should be ethically appointed by the patient to help understand the information provided, and it is not ethically appropriate for a doctor to skip a patient and obtain consent directly from the patient's relatives [19].

## 5 Study limitations

There were some limitations in our study that should be considered when interpreting the results. This study was self-funded, so it was only conducted in a single private hospital. Additionally, the number of patients was smaller than could be obtained by a multi-center study. Therefore, it is possible that a degree of unintentional selection bias was introduced by the single institution and smaller number of patients whose opinions were obtained. It would be useful for follow-up studies to be conducted in semi-government and government hospitals to obtain broader-based perceptions as the number of patients in the government hospitals in much higher and the doctor to patient ratio is also relatively less compared to private hospitals. So due to a higher number of patients in government hospitals there is less time to obtain a proper informed consent that might affect the various practices regarding the process of informed consent in those settings.

## 6 Conclusion

Our results showed that the patients had adequate knowledge about the process of informed consent. We identified language, better educational status, and not being asked by relatives as barriers to signing the consent form by the patients themselves. We also believe that it is imperative to involve the patients in the informed consent process and to ensure that they are given the opportunity either to sign the consent form themselves or approve of and observe the signing by their surrogate in the patient's presence.

## Ethical approval

Taken.

## Sources of funding for your research

None.

## Author contribution

MAA, ZA: Study concept or design. MAA, ZA: Data collection. NO: Data analysis or interpretation. MAA, ZA: Writing the first draft. KB, MI, IU: Revised draft. KB, MI, IU: Critical revision of the article.

## Registration of research studies

Name of the registry: Approved by the Institutional Review Board of our FMH College of Medicine and Dentistry hospital.
Unique Identifying number or registration ID: 07-2019-IRB-641-M
Hyperlink to your specific registration (must be publicly accessible and will be checked):

## Consent

NA.

## Guarantor

Muhammad Asharib Arshad.

FMH College of Medicine and Dentistry, Lahore Pakistan.

Email: asharibarshad@hotmail.com.

Irfan Ullah.

Kabir Medical College, Gandhara University.

Peshawar, Pakistan.

Irfanullahecp2@gmail.com.

## Provenance and peer review

Not commissioned, externally peer-reviewed.

## Declaration of competing interest

None.

## Data Availability

Data will be made available on request.
